# Alcohol and e-cigarette damage alveolar-epithelial barrier by activation of P2X7r and provoke brain endothelial injury via extracellular vesicles

**DOI:** 10.21203/rs.3.rs-3552555/v1

**Published:** 2023-11-14

**Authors:** Naveen Mekala, Jayshil Trivedi, Priyanka Bhoj, Namdev Togre, Slava Rom, Uma Sriram, Yuri Persidsky

**Affiliations:** Temple University; Temple University; Temple University; Temple University; Temple University; Temple University; Temple University

**Keywords:** Ethanol, electronic cigarette, pulmonary alveolar epithelial cells, P2X7r, A804598, extracellular vesicles, paracrine signaling

## Abstract

**Background:**

Use of nicotine containing products like electronic cigarettes (e-Cig) and alcohol are associated with mitochondrial membrane depolarization, resulting in the extracellular release of ATP, and mitochondrial DNA (mtDNA), mediating inflammatory responses. While nicotine effects on lungs is well-known, chronic alcohol (ETH) exposure also weakens lung immune responses and cause inflammation. Extracellular ATP (eATP) released by inflammatory/stressed cells stimulate purinergic P2X7 receptors (P2X7r) activation in adjacent cells. We hypothesized that injury caused by alcohol and e-Cig to pulmonary alveolar epithelial cells (hPAEpiC) promote the release of eATP, mtDNA and P2X7r in circulation. This induces a paracrine signaling communication either directly or via EVs to affect brain cells (human brain endothelial cells - hBMVEC).

**Methods:**

We used a model of primary human pulmonary alveolar epithelial cells (hPAEpiC) and exposed the cells to 100 mM ethanol (ETH), 100 μM acetaldehyde (ALD), or e-Cig (1.75μg/mL of 1.8% or 0% nicotine) conditioned media, and measured the mitochondrial efficiency using Agilent Seahorse machine. Gene expression was measured by Taqman RT-qPCR and digital PCR. hPAEpiC-EVs were extracted from culture supernatant and characterized by flow cytometric analysis. Calcium (Ca^2+^) and eATP levels were quantified using commercial kits. To study intercellular communication via paracrine signaling or by EVs, we stimulated hBMVECs with hPAEpiC cell culture medium conditioned with ETH, ALD or e-cig or hPAEpiC-EVs and measured Ca^2+^ levels.

**Results:**

ETH, ALD, or e-Cig (1.8% nicotine) stimulation depleted the mitochondrial spare respiration capacity in hPAEpiC. We observed increased expression of P2X7r and TRPV1 genes (3–6-fold) and increased intracellular Ca^2+^ accumulation (20–30-fold increase) in hPAEpiC, resulting in greater expression of endoplasmic reticulum (ER) stress markers. hPAEpiC stimulated by ETH, ALD, and e-Cig conditioned media shed more EVs with larger particle sizes, carrying higher amounts of eATP and mtDNA. ETH, ALD and e-Cig (1.8% nicotine) exposure also increased the P2X7r shedding in media and via EVs. hPAEpiC-EVs carrying P2X7r and eATP cargo triggered paracrine signaling in human brain microvascular endothelial cells (BMVECs) and increased Ca^2+^ levels. P2X7r inhibition by A804598 compound normalized mitochondrial spare respiration, reduced ER stress and diminished EV release, thus protecting the BBB function.

**Conclusion:**

Abusive drugs like ETH and e-Cig promote mitochondrial and endoplasmic reticulum stress in hPAEpiC and disrupts the cell functions via P2X7 receptor signaling. EVs released by lung epithelial cells against ETH/e-cig insults, carry a cargo of secondary messengers that stimulate brain cells via paracrine signals.

## Introduction

Electronic cigarettes (e-Cig) have gained popularity over combustible cigarettes, especially among young adults worldwide[1]and most of them are unaware that e-Cig vape contains higher nicotine concentration than combustible cigarettes does[2, 3]. Compared to combustible cigarette smokers and non-smokers, e-Cig smokers trigger early chronic inflammatory lung diseases [4]. Moreover, e-Cig vape induces the release of proinflammatory cytokines, including tumor necrosis factor alpha (TNF-α) and interleukin (IL)-1β, which promote chronic inflammation, pathologic changes in the lung parenchyma, and mitochondrial reactive oxygen species (ROS) buildup in lung epithelial cells[5, 6].

Although alcohol consumption is not directly linked to the onset of lung diseases, chronic alcohol exposure weakens lung responses to infections, particularly in the upper respiratory tract, resulting in poor immune response to pre-existing lung diseases or acquired infections[7–9]. Alcoholism is a major factor in the spread of community-acquired pneumonia and other acute respiratory complications, leading to thousands of deaths in the United States[10]. Excessive alcohol consumption alters mitochondrial structure[11] and reduces mitochondrial antioxidant glutathione levels, making mitochondria more susceptible to oxidative damage and ROS buildup, thus limiting ATP synthesis[12, 13]. Mitochondrial ROS accumulation instigates inflammation and DNA damage in lung epithelial cells[14]. Chronic alcohol exposure also stimulates purinergic P2X7 receptor (P2X7r), which activates NLRP3-mediated inflammasome in machrophages and releases extracellular ATP (eATP) as secondary messengers[15].

Lately, the association between inflammation and P2X7r has received much attention, with eATP release being a key source of inflammation[16, 17]. Furthermore, P2X7r activation promotes a sustained increase in intracellular calcium (Ca^2+^) levels, which increases endoplasmic reticulum (ER) stress, eventually leading to inflammation[18, 19]. Our prior studies have demonstrated the influence of addictive drugs [ethanol (ETH) and e-Cig vape] on P2X7r activation, which facilitated intracellular Ca^2+^ accumulation and eATP release in brain microvascular endothelial cells (BMVECs). Pretreatment with the P2X7r antagonist A804598 (A80) restored homeostasis, prevented the blood-brain-barrier (BBB) compromise[20].

P2X7r activation by eATP also stimulates the generation of heterogeneous extracellular vesicles (EVs) that carry biomolecular cargoes that can mediate communication between similar (endocrine signaling) or different (paracrine signaling) cell types [21, 22]. EVs are bilayered membrane vesicles formed in an endosomal system and discharged into the extracellular space[23]. Although the cargo-carrying capacity of EVs is well established, the composition of the cargo does not always reflect the contents of the parental cells. Cells incorporate cargo into the EVs in a carefully controlled manner, reflecting the pathophysiological state of the cell[24]. During alcohol abuse, hepatocyte EVs transport broken mitochondrial DNA (mtDNA), which act as damage-associated molecular patterns (DAMPs), triggering inflammatory responses[25]. Similarly, EVs in patients with fatty liver conditions carry higher mtDNA and stimulate the innate immune response via the TLR-9 pathway[26]. In the past, the existence of diverse messengers, including proteins, lipids, nucleic acids, and cell organelles, has been well identified and characterized as EV cargo[27, 28], while data on eATP content as secondary messengers are sparse.

In our earlier study, we showed the effects of ETH and e-Cig on P2X7r regulation in BMVECs[20]. In this report, we assessed the neuroinflammatory effects of ETH and e-Cig vape on primary human pulmonary alveolar epithelial cells (hPAEpiC) via EVs. We specifically analyzed the effectiveness of P2X7r blockage by A80 on responses to e-Cig and ETH exposure, focusing on mito-stress regulation in hPAEpiC. We also examined its effect on intracellular Ca^2+^ accumulation, size and quantity of EVs released by the hPAEpiC. Finally, we examined the presence of P2X7r in the EVs, measured the eATP- and mtDNA-carrying capacity of hPAEpiC EVs, and tested their potential to mediate long-distance communication between hPAEpiC and BMVECs.

## Materials and Methods

### Reagents/Kits

ETH 200 Proof, 99.5% pure aldehyde (ALD), and e-Cig (1.8% and 0% nicotine) were procured from Decon Laboratories Inc. (Cat. No. 2716, King of Prussia, PA, USA), ACROS Organics (Cat. No. 402788, Geel, Belgium), and Pure E-Liquids (Peterborough, PE11SB, UK), respectively. A80 was purchased from Tocris Bioscience (Cat. No. 4473, Bristol, UK) and dissolved in DMSO from Sigma-Aldrich (Cat. No. D5879, St. Louis, MI, USA). We purchased the luminescent ATP detection and colorimetric Ca^2+^ assay kits from Abcam (Cat. Nos. ab113849 and ab102505, respectively, Cambridge, UK). For EV isolation and capture, we used the ExoQuick-TC from SBI (Cat.No. EXOTC50A, Palo Alto, USA) and Tetraspanin Exo-Flow capture kit (Cat. No. EXOFLOW150A-1, Palo Alto, USA), respectively. anti-P2X7r antibodies (Alomone Labs, Jerusalem, Israel), were labelled using the Zip Alexa Fluor^™^ 647 rapid antibody labeling kit from Invitrogen (Cat. No. Z11235, Waltham, USA). The primary anti-rabbit antibodies for p-IRE1 (Cat. No. 3294T), p-ASK1 (Cat. No. 3765S), CD9 (Cat. No. 13174S), CD81 (Cat. No. 56039S), and beta-actin (Cat. No. 4967S) were procured from Cell Signaling (Danvers, MA, USA). Anti-rabbit Bax inhibitor-1 [BI-1] (Cat. No. ab18852) polyclonal antibodies were procured from Abcam (Cambridge, UK). Human purinergic receptor P2X, Ligand Gated Ion Channel 7 (P2RX7) ELISA Kit (Cat. No. RDR-P2RX7-Hu) obtained from Reddot Biotech (Houston, USA). Lyophilized recombinant human P2X7r protein (Cat. No. LS-G25681–10) procured from LS Bio (Lynnwood, USA).

### Cell cultures and treatments

hPAEpiC, acquired from Accegen (Cat. No. ABC-TC3770, Fairfield, USA), were cultured in hPAEpiC basal media (Cat. No. ABM-TM3770), supplemented with insulin-transferrin-selenium, epidermal growth factor, hydrocortisone, and 5% fetal bovine serum. hBMVECs, provided by Dr. Pierre-Olivier Couraud, Institut Cochin, INSERM U1016, CNRS UMR 8104, Université Paris Descartes (Paris, France), [29] were cultured using EBM-2 basal medium (Cat. No. CC-3156), supplemented with EGM-2 SingleQuots (Cat. No. CC-4176) from Lonza Biosciences (St. Bend, USA). According to the experiment, both hPAEpiC and hBMVECs were cultured in 96-well plates, 6-well plates, 100-mm tissue culture plates, and T-75 and T-150 flasks, coated with bovine collagen from CELL applications. Inc (Cat. No. 123 − 100, San Diego, USA). Confluent hPAEpiC were pretreated with and without A80 (10 μM) for 1 h, followed by overnight stimulation with insults: ETH-(100 mM), ALD-(100 μM), or e-Cig (1.75μg/mL of 1.8% and 0% nicotine)-conditioned media. A80 treated cells served as a P2X7r antagonist control. Unless mentioned, all experiments were carried out after overnight incubation.

### Mito-stress analysis

hPAEpiC (20,000 cells/well) were plated in a seahorse XF96 microplate from Agilent Technologies (Cat. No. 103794–100, Santa Clara, USA) and allowed to attach overnight. Four corner wells were left empty for background correction. The next day, the cells were treated with A80 (10 μM) for 1 h, followed by replacing old media with growth media conditioned with ETH, ALD, and e-Cig (1.8% or 0% nicotine) for overnight, with and without A80. The next morning, mitochondrial stress was measured using the Agilent Seahorse Cell Mito Stress Test Kit (Cat. No.103015–100, Santa Clara, USA). In brief, the growth media was carefully aspirated, and cells were washed twice in bicarbonate-free and phenol red-free DMEM, from Agilent (Cat. No. 103680–100, Santa Clara, USA), supplemented with 5.5 mM glucose, 1 mM pyruvate, and 2 mM glutamine. Lastly, 180 μL DMEM was added in all 96 wells including the four corner wells, and cells were kept in a non-CO_2_ incubator at 37°C. After calibration of Seahorse cartridge, microplate was placed in the Seahorse analyzer, and basal oxygen consumption rates (OCR) were measured. Later, the cells were serially challenged with respiratory inhibitors-2.5 μM oligomycin (ATP synthase inhibitor), 0.5 μM FCCP (mitochondrial uncoupler), and 0.5 μM rotenone/antimycin A (complex I/III inhibitor) and mitochondrial respiration levels were continuously recorded. After the assay, the spare respiratory capacity (SRC) was measured by subtracting the basal OCR from the maximal OCR.

### Quantitative RT-PCR

Total RNA from hPAEpiC was isolated using the TRIzol^™^ reagent from Invitrogen (Cat. No. 15596026, Carlsbad, USA). Total RNA (400 ng) was converted into complementary DNA (cDNA) using the RT2 PreAMP cDNA Synthesis Kit (Cat. No. 330451, Qiagen, Germantown, MD, USA). TaqMan probes for human P2X7r (Cat. No. hs00175721), transient receptor potential vanilloid 1 (TRPV1; Cat. No. hs00218912), and GAPDH (Cat. No. hs02786624) were procured from Thermo Fisher (Waltham, USA). The cDNA was further probed for real-time qPCR using TaqMan Fast Advanced Master Mix (Cat. No. 4444557, Applied Biosystems, Waltham, USA). All reactions were performed in triplicate, and the relative fold-change of the P2X7r and TRPV1 gene expressions against the treatments were investigated using delta-delta-Ct (ddCt), and the values were normalized with ddCt values of GAPDH.

### Intracellular Ca^2+^ analysis

Confluent hPAEpiC, pre-treated with or without A80, were stimulated overnight with ETH, ALD, and e-Cig (1.8% or 0% nicotine) conditioned media, and intracellular Ca^2+^ levels were measured using Abcam Ca^2+^assay kit following the manufacturer’s instructions.

### Western blot analysis

Denatured proteins from hPAEpiC were separated using SDS-polyacrylamide gels (4–20% Mini-PROTEIN TGX^™^ Precast Gels) and electroblotted onto nitrocellulose membranes. The membranes were blocked (1 h) with Intercept blocking buffer from LI-COR (Cat. No. 927–60001, Lincoln, USA), incubated overnight with primary antibodies (1:1000 dilution), probed with near-infrared secondary antibodies (LI-COR) (1:5000 dilution), and visualized using an Odyssey imaging system (LI-COR, Lincoln, USA). Band intensities were quantified, and protein expression levels were analyzed relative to beta-actin.

### EV isolation

After the overnight stimulation of hPAEpiC with the insults in T-150 flasks, 25 mL culture supernatant was collected in 50 mL Falcon^™^ tubes from Fischer Scientific (Cat. No. 14–959-49A, Hampton, USA) and centrifuged at 2,000g for 10 min to remove cell debris. Supernatant was transferred into a fresh 50 mL tube and further centrifuged at 10,000g for 20 min to remove apoptotic bodies and other large particles from the media. The supernatant was further concentrated (5 mL–7.5 mL) using Amicon^®^ Ultra-15 centrifugal filters from MilliporeSigma (Cat. No. UFC901024, Burlington, USA). Appropriate volume of ExoQuick-TC^™^ was added to the media (1 mL per 5 mL media), and the contents were mixed thoroughly by inverting the tubes, followed by overnight refrigeration at 4°C. Next morning, contents were centrifuged at 2000g for 20 min at 4°C, and the EV pellet was resuspended in sterile phosphate-buffered saline (PBS) without calcium and magnesium from MilliporeSigma (Cat. No. D8537, Burlington, USA).

### Nanoparticle tracking analysis of EVs

The number and particle size distribution of hPAEpiC-EVs were analyzed by nanoparticle tracking analysis (NTA) using the NanoSight NS300 system (Malvern Technologies, Malvern, UK) fixed with a 488 nm laser. EV samples were diluted (1:500) in 1 mL particle-free MilliQ water (Milliporesigma, Burlington, USA) and injected into NanoSight chamber using 1 mL BD slip-tip syringe (Cat. No. 309659, Franklin Lakes, USA). Sample analysis was carried out under constant particle flow into the NanoSight chamber, and five 30-second videos were recorded for each sample. These videos record and track the path of unlabeled particles/EVs acting as point scatterers, undergoing Brownian motion in the chamber using laser beam [30]. Data collected in this fashion was later analyzed by NTA 3.3.104 software. Before running the samples, 100 nm latex beads from Malvern (Cat. No. NTA4088) were used to calibrate the system.

### eATP detection in isolated EVs

hPAEpiC-EVs were resuspended in 150 μL PBS (Ca^2+^ and Mg^2+^ free) and lysed by ultrasonication at 4°C. The EV suspension was centrifuged at 10,000g for 10 min, and 50 μL sample was loaded in duplicates in the Corning^®^ black clear bottom 96-well plate (Cat. No. 3603, Corning, USA). Abcam luminescent ATP Detection assay kit was used to measure eATP cargo in EVs by following the manufacturer’s instructions.

### DNA isolation from EVs

The EV suspension (100 μL) was treated with 10U of DNase from LGC Biosearch Technologies (Cat. No. DB0715K, Hoddesdon, UK) for 20 min at 37°C to eliminate DNA attached to the EV surface. The DNase action was stopped by adding 10 μL 10X DNase stop solution at 65°C for 10 min. EVs in suspension were further diluted by adding 100 μL nuclease-free water (NFW) and lysed by adding 20 μL proteinase K from Thermo Fisher (Cat. No. 4485229, Waltham, USA) at room temperature. After this step, the DNeasy^®^ Blood & Tissue kit from Qiagen (Cat. No. 69506, Hilden, DE) was used to isolate DNA from EVs. We followed the manufacturer’s instructions for DNA isolation, except for centrifugation at 20,000g for 1 min after the addition of AW2 buffer [31]. Similarly, spin columns were preincubated in AE buffer (30 μL) for 5 min before DNA elusion at room temperature. Remaining DNA in spin columns was also eluted by introducing an additional spin step. EV-DNA was quantified and stored at −20°C prior to digital PCR (dPCR) assay.

### mtDNA quantification by dPCR

A working concentration (1 ng/μL) of EV-DNA samples was prepared in NFW. Mitochondrial gene-specific Taqman^™^ probes for ATP8 [mt-ATP8] (Cat. No. 4331182 Hs02596863_g1), NADH dehydrogenase 2 [mt-ND2] (Cat. No. 4331182 Hs02596874_g1), and ferritin heavy chain 1 [mt-FTH1](Cat. No. 4331182 Hs02596865_g1) from Thermo Fisher Scientific (Waltham, USA) were used in dPCR experiments. For 10 μL dPCR reaction, we used 2 μL of 5X Absolute Q^™^ DNA Digital PCR Master Mix (Cat. No. A52490), 2 μL EV-DNA template (2 ng), 0.5 μL FAM-Taqman^™^ probe, and 5.5 μL NFW. Nine microliters of the above reaction mixture were loaded onto QantStudio^™^MAP16 Digital PCR plate (Cat. No. 10246917). Lastly, 15 μL QuantStudio^™^ Isolation buffer (Cat. No. A52730) was added on top of each sample, and the wells were sealed with the gaskets supplied with the dPCR plates. The QuantStudio^™^ Absolute Q Digital PCR System from Thermo Fisher was used for DNA amplification, and QuantStudio dPCR software was used to count the number of microchambers with successful mtDNA amplification. The thermal profile of mtDNA dPCR was as follows: 10 min at 96°C, followed by 40 cycles of 5 sec at 96°C and 15 sec at 60°C.

### P2X7r ELISA

Cell culture supernatants collected from insult-stimulated hPAEpiC, with and without A80 treatment, were used to detect circulating P2X7r levels. Human purinergic P2RX7 ELISA Kit was used for this assay. In brief, 200 μL medium was added in appropriate wells, covered with a plate sealer, and incubated at 37°C for 90 min. Culture media was removed from the wells and replaced with one hundred microliter detection solution ‘A’ followed by 45 min incubation at 37°C. The wells were washed thrice with 300 μL 1X wash buffer. One hundred microliter detection solution ‘B’ was added to the wells and incubated at 37°C for 45 min. The washing step was repeated as mentioned earlier, and 90 μL ‘substrate solution’ was added to the wells. The ELISA plate was incubated at 37°C in the dark until a blue color developed in the wells (for 15–30 min). The enzymatic reaction was stopped by adding 50 μL stop solution. Absorbance was measured at 450 nm using a microplate reader (SpectraMax^®^ M5).

### Flow cytometry analysis of EVs

Magnetic streptavidin beads were conjugated with tetraspanin-coupled, biotinylated anti-CD9 or anti-CD63 antibodies provided in the Tetraspanin Exo-Flow Combo Capture Kit (System Biosciences, Palo Alto, USA). These magnetic beads were incubated with EV suspension overnight on a rotating mixer at 4°C. During this step, EVs were captured on to the conjugated magnetic beads. The next morning, magnetic beads were washed thrice in 1X wash buffer to remove any unbound EV particles. EVs captured on to magnetic beads were resuspended in 500 μL wash buffer and incubated with 5 μg of anti-P2X7r antibody conjugated with Alexa Fluor^™^ 647 overnight on a rotating mixer at 4°C. The magnetic beads were washed thoroughly to eliminate unbound P2X7r antibodies. EVs were stained with exo-FITC dye (System Biosciences). Cytometric acquisition was performed using an Aurora flow cytometer (Cytek^®^, San Diego, USA) and analyzed using FlowJo software v10 (Tree Star Inc., Ashland, USA) to check the distribution of P2X7r on EVs.

### Intracellular Ca^2+^ analysis in hBMVECs after EV stimulation

Intracellular Ca^2+^ levels in hBMVECs were measured after overnight incubation with cell culture supernatant or EVs from hPAEpiC. Confluent hBMVECs cultured in their native growth media were stimulated with freshly collected hPAEpiC supernatant conditioned in ETH, ALD and e-Cig (1.8% or 0% nicotine), with and without A80 pre-treatment. hBMVECs incubated with fresh hPAEpiC-cultured media were used as media control. In another experiment, hBMVECs cultured in 12-well plate (4 ×10^5^) were incubated with freshly isolated hPAEpiC-EVs (1:300). After 5 h incubation with EVs, intracellular Ca^2+^ levels in hBMVECs were measured using the calcium assay kit. Optimal condition for EV number and incubation time were determined from preliminary experiments (Supplementary figure S1). During this experiment, hBMVECs were never exposed with either insults or A80 directly.

## Results

### 1. P2x7r inhibition normalized mitochondrial oxidative phosphorylation (OXPHOS) in insult-exposed hPAEpiC

**Figure 1:**
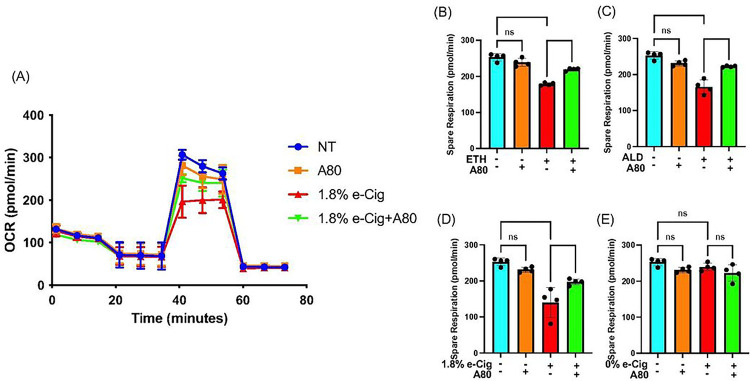
P2X7r inhibition prevented mitochondrial dysfunction, induced by ETH, ALD, or e-Cig (1.8% nicotine). After overnight exposure to insults, mito-stress levels in hPAEpiC were measured using a Seahorse analyser, and the SRC were calculated. In figure, **(A)** line graph depicts the spare respiration capacity (SRC), whereas Fig **(B), (C),** and **(D)** presents a 30%, 35%, and 42% reduction in spare respiration against 100 mM ETH, 100 mM ALD, and 1.75mg/mL e-Cig (1.8% nicotine) stimulation, respectively. **(E)** e-Cig extract with 0% nicotine had no impact on mitochondrial SRC. Pre-treatment with A80 (10 mM) restored OXPHOS levels in the insult-exposed hPAEpiC. Data were normalized to untreated control cells, and one-way ANOVA was used for statistical analyses, *P≤ 0.05, ****P ≤ 0.0001, and ns (not significant) (n=4).

hPAEpiC exposed with ETH-, ALD-, and e-Cig (1.8% nicotine)-conditioned media (same concentrations as physiological levels in humans) increased the mito-stress levels in cells, resulting in reduced SRC levels as shown in line graph (Fig. 1A). Whereas, Fig. 1B–D illustrate 30–42% reduction in the SRC against ETH, ALD, and e-Cig (1.8% nicotine) exposure. Importantly, A80-pretreated hPAEpiC were protected from insult-driven mitochondrial stress, with restored SRC.

### 2. Increased P2X7r and TRPV1 channel expression led to Ca ^2+^ accumulation in the insult-exposed hPAEpiC.

**Figure 2:**
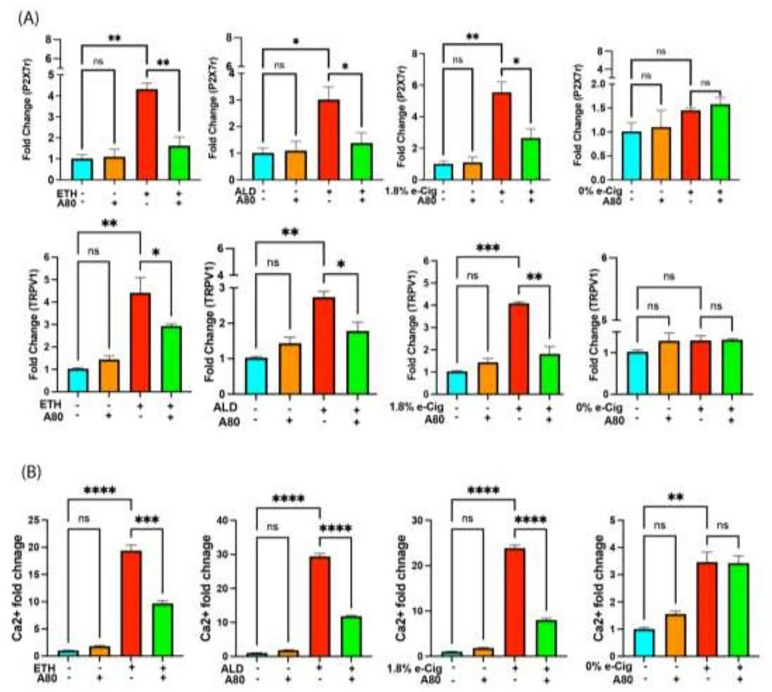
ETH, ALD, and e-Cig (1.8% nicotine) increased P2X7r and TRPV1 expression and Ca ^2+^ accumulation in hPAEpiC. **(A)** Overnight stimulation with ETH-, ALD-, and e-Cig (1.8% nicotine)-conditioned media increased P2X7r and TRPV1 gene expression by 4–6-fold and 3–4-fold, respectively. A80 pretreatment significantly lowered the P2X7r and TRPV1 expression in cells. e-Cig (0% nicotine)-conditioned media had no significant effect on P2X7r and TRPV1 expression in hPAEpiC. qPCR data were normalized to untreated cells, and GAPDH was used as a housekeeping gene. **(B)** Inhibition of P2X7r and TRPV1 expression by A80 reduced Ca^2+^ accumulation in insult-treated hPAEpiC. Overnight stimulation with ETH-, ALD-, and e-cig (1.8% nicotine)-conditioned media increased intracellular Ca^2+^ levels by 20–30-folds. A80 pretreatment significantly reduced the levels. Data were normalized with untreated control cells. One-way ANOVA was used for statistical analyses. *P ≤ 0.05, **P ≤ 0.01, ***P ≤ 0.001, ****P ≤ 0.0001, and ns (not significant) (n=3).

After the overnight exposure of hPAEpiC to the insults, P2X7r and TRPV1 expression increased by 4–6-fold and 3–4-fold, respectively. A80 pretreatment significantly lowered the overexpression of both channels (Fig. 2A). e-Cig with 0% nicotine had no significant impact on the expression levels of P2X7r and TRPV1 channels. Furthermore, we found a 20–30-fold increase in intracellular Ca^2+^ accumulation in the insult-exposed hPAEpiC compared with untreated control cells. Interestingly, e-Cig with 0% nicotine also increased intracellular Ca^2+^ levels by 4-fold, which was a non-acute buildup and did not impact other functional readouts. A80 pretreatment significantly decreased the intracellular Ca^2+^ levels in the insult-exposed HPAEpiC (Fig. 2B).

### 3. Alteration in Ca ^2+^ homeostasis upregulated the ER stress in the insult-exposed hPAEpiC.

**Figure 3:**
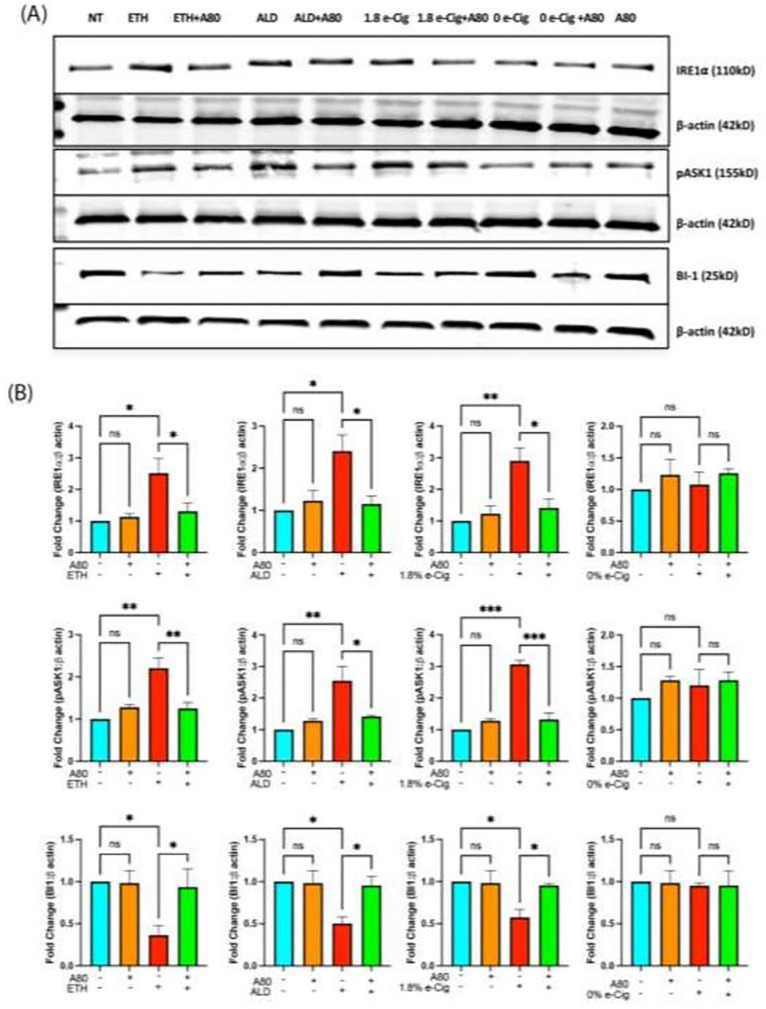
P2X7r inhibition prevented ER stress that was escalated by ETH, ALD, or e-Cig (1.8% nicotine) in hPAEpiC. (A) Representative western blot image from one of three independent experiments (B) Bar graphs show 2 to 3-fold increase in the expression of the ER stress markers IRE1 and pASK1, while the expression of the antiapoptotic Bax inhibitor-1 (BI-1) protein was cutdown by 50%, resulting in the amplification of mito-stress levels in hPAEpiC. A80 diminished pro-apoptotic protein expression and restored antiapoptotic protein expression. Western blot data were normalized to β-actin. One-way ANOVA was used for statistical analyses, *P ≤ 0.05, **P ≤ 0.01, ***P ≤ 0.001, and ns (not significant) (n=3).

Transient stimulation of P2X7r and TRPV1 is known to facilitate Ca^2+^ influx into cells, stimulating diverse pro/anti-apoptotic pathways in a cell-specific manner[32, 33]. Unrestricted Ca^2+^ influx into the cytosol often interferes with ER Ca^2+^ levels, as most ER-localized chaperones depend on Ca^2+^ ions for their function. Disruption in ER Ca^2+^ levels causes protein aggregation, followed by unfolded protein response (UPR)[34]. The UPR promotes the phosphorylation of ER-specific, pro-apoptotic inositol-requiring enzyme 1 alpha (IRE1α) and its downstream regulator, apoptosis signal-regulating kinase (ASK1) MAP3K, forcing cells to undergo apoptosis[20, 35]. Using western blotting, we showed that hPAEpiC exposed with ETH-, ALD-, or e-Cig (1.8% nicotine)-conditioned media increased the phosphorylation of IRE1α and ASK1 by 2 to 3-fold, respectively. Simultaneously, the expression of anti-apoptotic protein Bax inhibitor-1 (BI-1) was down-regulated by 50%, potentially stimulating the apoptosis of hPAEpiC (Fig. 3). A80 pre-treatment reversed the expression level of ER stress markers in insult-exposed HPAEpiC.

### 4. Enhanced ER stress after ETH, ALD, or e-Cig (1.8% nicotine) stimulation increased lung epithelial EV numbers and particle-size.

**Figure 4:**
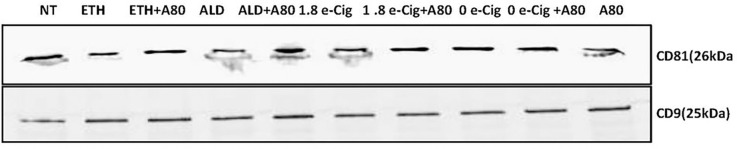
EVs were further evaluated for the presence of CD81 and CD9 markers using western blot.

UPR stimulated by alcohol and other abusive drugs facilitates ER stress, followed by ER-Ca^2+^ efflux into the mitochondrial matrix, resulting in mitochondrial OXPHOS reduction and ROS accumulation[36, 37]. Mitochondrial dysfunction and ROS buildup promote EV release in mouse myeloblast cells[38]. Similarly, morphine-exposed BMVECs revealed redox imbalance, resulting in unwarranted EV release[39]. In this context, hPAEpiC exposed with ETH, ALD, and e-Cig (1.8% nicotine) conditioned media increased the EV release by 2 to 3 folds and average size of EVs were inflated by 20–30%. Pretreatment of hPAEpiC with A80 significantly reduced the EV number (Table. 1). We further confirmed the tetraspanin profile (CD81 and CD9 expression) of isolated EVs by western blots (Fig. 4).

### 5. Larger EVs carried more eATP and mtDNA

**Figure 5:**
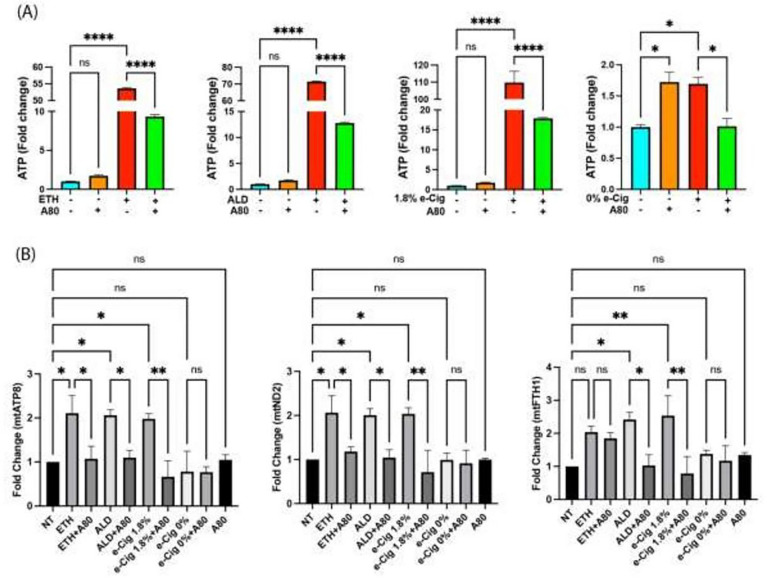
ETH, ALD, or e-Cig (1.8% nicotine) stimulation facilitated eATP and mtDNA buildup in hPAEpiC-EVs. **(A)** In isolated EVs, both ETH and ALD stimulation increased the eATP levels by 55-fold and 70-fold, respectively, whereas e-Cig (1.8% nicotine) stimulation elevated the eATP levels by 110-fold. EVs isolated from hPAEpiC pretreated with A80 showed significantly lower eATP levels. Although, eATP levels in e-Cig (0% nicotine)-treated cells are significant, fold changes were small compared to other insults. **(B)** dPCR was used to quantify the copy numbers of mtDNA present in EVs, and bar graphs represent the fold changes in the experimental groups, normalized to EVs from untreated control. We used 2 ng of EV DNA for dPCR amplification. Overnight stimulation with ETH, ALD, and e-Cig (1.8% nicotine) doubled the mtDNA cargo, whereas A80 pretreatment could successfully block the mtDNA assembly in EVs. One-way ANOVA was used for statistical analyses, *P ≤ 0.05, **P ≤ 0.001, ****P ≤ 0.0001, and ns (not significant) (n=3).

Abusive drugs like cigarette smoke and alcohol are known to influence the EV cargo in liver and lung cells [40, 41]. In this direction, we measured eATP levels in isolated EVs after overnight stimulation of hPAEpiC with ETH-, ALD-, or e-Cig-conditioned media. ETH and ALD induced a 55-fold and 70-fold increase in eATP levels, respectively, while e-Cig (1.8% nicotine) stimulation resulted in a 110-fold increase. Although e-Cig (0% nicotine) stimulation increased the eATP levels in EVs, a 2-fold change was insufficient to influence downstream events. Simultaneous pretreatment with A80 significantly lowered eATP levels in hPAEpiC-EVs (Fig. 5A).

We used high-throughput dPCR to measure the absolute copy numbers of mtDNA in the EVs using Taqman^™^ probes targeting various segments of the mtDNA. Overnight stimulation with ETH-, ALD-, or e-Cig (1.8% nicotine)-conditioned media increased the copies of mt-ATP8, mt-ND2, and mt-FTH1 in the EVs by 2-fold, whereas pretreatment with A80 effectively diminished the mtDNA cargo (except mt-FTH1 levels after ETH stimulation) (Fig. 5B). e-Cig (0% nicotine) did not show significant changes in the mtDNA levels when compared to the untreated control group. We presented dPCR data in fold change to show statistical significance between experimental replicates.

### 6. ETH, ALD, or e-Cig (1.8% nicotine) exposure promoted P2X7r shedding via cell supernantant and EVs.

**Figure 6:**
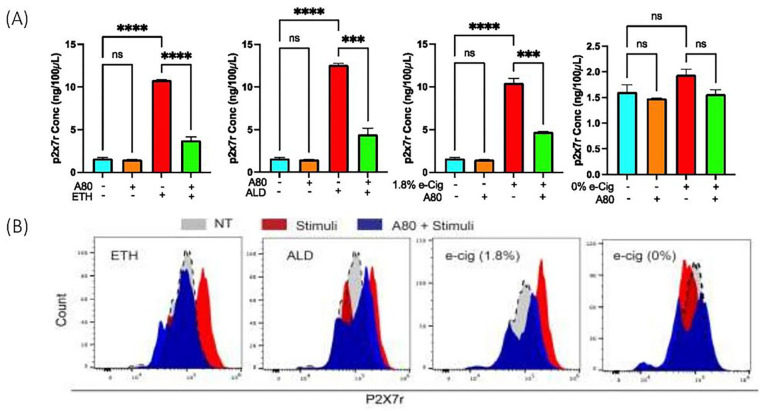
ETH, ALD, or e-Cig (1.8% nicotine) exposure increased the circulating P2X7r levels both in culture supernatant and on the EV surface. **(A)** ETH or e-Cig (1.8% nicotine) stimulation resulted in a 6-fold increase in circulating P2X7r levels, and ALD stimulation elevated circulating/soluble P2X7r level by 8-fold. Media from A80 pretreated, insult-exposed cells showed significantly lower P2X7r levels than media with only insult-exposed cells. One-way ANOVA was used for statistical analyses; ***P ≤ 0.001 ****P ≤ 0.0001, and ns (not significant) (n=3). **(B)** EVs captured on to the magnetic beads presented increased MFI values (P2X7r distribution) after ETH (1.6-fold), ALD (1.5-fold), and 1.8% e-Cig stimulation (1.53-fold), and pretreatment with P2X7r antagonist (A80) successfully reduced the MFI values.

In hPAEpiC supernatant,ETH and e-Cig (1.8% nicotine) stimulations elevated the P2X7r levels by 6-fold. ALD exposure led to an 8-fold increase in P2X7r levels. Cell supernatant from A80-pretreated cells had significantly lower P2X7r levels compared to insult-exposed cells. e-Cig (0% nicotine) had no effect on P2X7r shedding (Fig. 6A).

Flow cytometric analysis of EVs was performed to assess the potential distribution of P2X7r on the EV surface. ETH, ALD, and e-Cig (1.8% nicotine) stimulations increased median fluorescence intensity (MFI) by 50 to 60%, showing greater P2X7r cargo on the EVs compared with the EVs from the unexposed-cells. EVs isolated from A80-pretreated, insult-exposed cells showed lower P2X7r MFI than only insult-exposed cells. E-Cig (0% nicotine) stimulation showed no effects on MFI (Fig. 6B).

### 7. Soluble P2X7r and eATP released from hPAEpiC-EVs induce paracrine signaling in BMVECs

**Figure 7:**
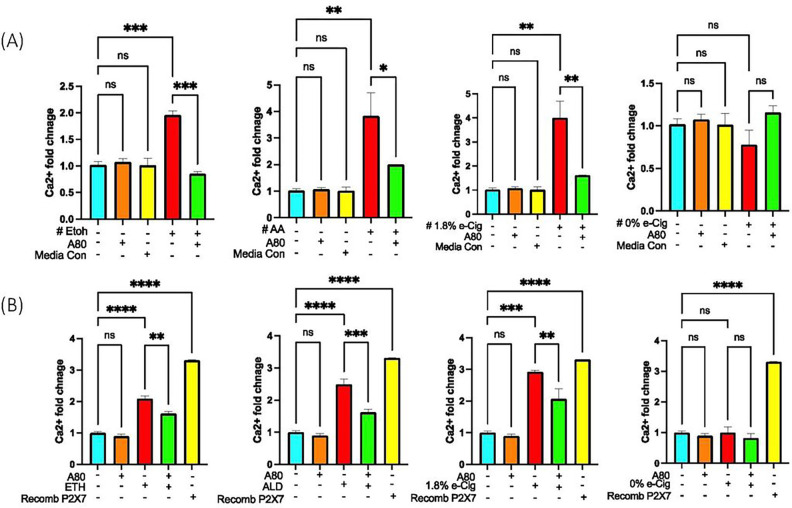
EVs containing P2X7r and eATP cargo mediated intercellular signaling between hPAEpiC and hBMVECs. **(A)** Conditioned epithelial cell culture media with ETH induced a 2-fold increase in Ca^2+^ levels in BMVECs, and 4-fold increase against ALD or e-Cig (1.8% nicotine) conditioned media. BMVECs treated with media preconditioned with A80 could successfully block the intracellular Ca^2+^ accumulation. BMVECs incubated with lung epithelial cell media were used as a media control. **(B)** Lung epithelial EVs shed against ETH, ALD, and e-Cig (1.8% nicotine) stimulation were incubated (5 h) with BMVECs (300 EVs/cell). Lung EVs increased the BMVEC’s Ca^2+^ levels 2 to 3-fold. Human recombinant P2X7r protein expressed in the prokaryotic system (500ng/ml) from LS bio was used as a positive control. One-way ANOVA was used for statistical analyses, *P ≤ 0.05, **P ≤ 0.01, ***P ≤ 0.001, ****P ≤ 0.0001, and ns (not significant) (n=3).

Extracellular ATP-gated P2X7r activation stimulates Ca^2+^ influx into the cytosol, activating inflammasome assembly and caspase-1 [42]. Recently, circulating P2X7r was shown to trigger inflammasome formation in the brain of epilepsy patients[43]. In our studies, we examined the paracrine signaling between hPAEpiC and human BMVECs by culturing BMVECs in hPAEpiC-conditioned media. After overnight stimulation, intracellular Ca^2+^ levels were measured in BMVECs. Media from ETH-exposed hPAEpiC increased the BMVEC’s Ca^2+^ levels by 2-fold, whereas ALD or e-Cig (1.8% nicotine)-conditioned media escalated the BMVEC’s Ca^2+^ levels by 4-fold. Media preconditioned with A80 had considerably reduced intracellular Ca^2+^ levels in BMVECs (Fig. 7A). BMVECs incubated in media from unexposed-hPAEpiC were used as a media control.

To prove the specific effects of the signaling abilities of lung EVs, we incubated BMVECs with freshly isolated lung EVs (300 EVs/cell). After 5 h incubation, intracellular Ca^2+^ levels were measured using a calcium assay. EVs isolated after ETH, ALD, and e-Cig (1.8% nicotine) stimulation amplified the intracellular Ca^2+^ levels by 2–3-fold, whereas EVs from A80-pretreated cells presented lower Ca^2+^ levels (Fig. 7B). Of note, recombinant P2X7r (used as a control) also increased Ca2 + accumulation in BMVECs, establishing the functional role of P2X7r as a Ca2 + channel.

## Discussion

In this report, we studied the harmful effects of alcohol, e-Cig vaping, and their byproducts on mitochondrial homeostasis in hPAEpiC, EV shedding, and EV cargo content. In mitochondria, respiratory capacity depends on the efficiency of electron transport complexes and mitochondrial membrane potential[44]. In the liver, chronic alcohol consumption and cigarette smoke accelerate reactive oxygen and nitrogen species (ROS/RNS) accumulation through NADPH oxidase (NOX) and cytochrome P450–2E1 (CYP2E1) enzyme activation[45]. In an intracerebral hemorrhage mouse model, P2X7r activation is shown to mediate NOX-dependent ROS production, followed by mitochondrial degradation[46]. NOX is a catalytic enzyme that transfers electrons (e^−^) from NADPH to oxygen, generating superoxide radicals (O_2_^•−^)[47]. CYP2E1 is an ethanol-catabolizing enzyme, known for ROS/RNS generation in significant amount[48]. Upon ROS/RNS buildup, the inner mitochondrial membrane quickly depolarizes and limits the OXPHOS levels[49]. In our Seahorse experiments, hPAEpiC exposed with ETH-, ALD-, or e-Cig-conditioned media significantly reduced the OXPHOS levels, confirming the detrimental effects of abusive drugs on mitochondrial function (Fig. 1). Also, P2X7r inhibition restored the mitochondrial OXPHOS levels, confirming the role of P2X7r in regulating mitochondrial health in hPAEpiC.

P2X receptors are a family of ligand-gated ion channels, gated by eATP, and exist in seven isoforms, P2X1 to P2X7 receptors[50]. Unlike other P2X receptors, P2X7r needs excess ATP for its activation (three ATP molecules for one P2X7r). Activated P2X7r known to regulate Ca^2+^ and sodium (Na^+^) influx and potassium (K^+^) efflux in cells[51], mediate actin and tubulin rearrangement[52], promote inflammation[53], and encourages mitochondrial swelling/rupture to release pro-apoptotic cytochrome C into the cytosol[54]. While the involvement of P2X7r in various pathophysiological conditions is well reported, its potential role in substance abuse attracted attention only recently[55, 56]. Similarly, TRPV1 is a highly selective Ca^2+^ channel, which facilitates cigarette smoke-associated airway inflammation[57] and opioid-induced hyperglycemia[58]. In our studies, ETH, ALD, and e-Cig (1.8% nicotine) stimulation increased the gene expression of both P2X7r and TRPV1, enhancing Ca^2+^ influx into the hPAEpiC (Fig. 2). Such a sudden increase in intracellular Ca^2+^ levels can promote cytoskeletal remodeling[59], alter Ca^2+^ levels in the ER lumen, and activate Ca^2+^-dependent ER stress, leading to cell death[20]. As expected, the P2X7r inhibitor A80 successfully curbed the P2X7r and TRPV1 overexpression and reduced the Ca^2+^ influx into the hPAEpiC.

ER stores the large amount of Ca^2+^ with a steep concentration gradient between the ER (up to 1 mM) and cytosol (approximately 100 nM)[60]. ER-Ca^2+^ levels are vital for the post-translational modifications of transmembrane proteins in the ER lumen[34]. Cytosolic Ca^2+^ acts as an intracellular messenger that controls diverse cellular functions, and any disruption in cytosolic Ca^2+^ homeostasis can be toxic and cause cell death[61]. In our experiments, the P2X7r and TRPV1 overexpression significanly increased the intracellular Ca^2+^ levels in hPAEpiC, exposed to ETH, ALD, or e-Cig (1.8% nicotine). Under these pathological conditions, excess Ca^2+^ can be shuttled and stored in the ER by the energy-consuming sarcoplasmic/endoplasmic reticulum calcium ATPase (SERCA2b)[62]. SERCA2b overexpression during chronic inflammation promotes dramatic Ca^2+^ uptake by the ER, resulting in increased UPR in the ER[63].

IRE1α is an evolutionarily conserved ER membrane protein involved in the regulation of both cell survival and death mechanisms[64]. As discussed earlier, most secretory proteins are produced in the ER lumen and ER-Ca^2+^ levels are vital for proper protein folding[65]. Any fluctuations in ER-Ca^2+^ levels lead to protein misfolding, followed by UPR, which serve as direct ligands for IRE1α activation[66]. Its prolonged activation triggers the apoptosis-inducing molecule, tumor necrosis factor receptor-associated factor 2 (TRAF2), through its cytosolic domain. This further activates its downstream pASK1, a MAP kinase kinase kinase (MAP3K), which later phosphorylates c-Jun N-terminal kinase and p38, leading to apoptotic cell death[67, 68]. On the contrary, BI-1 plays a protective role against ER-Ca^2+^ buildup. BI-1 facilitates Ca^2+^ flow from the ER into the mitochondrial matrix via the mitochondrial permeability transition pore, thereby restoring Ca^2+^ levels in the ER lumen[69, 70]. In the present study, Ca^2+^ influx triggered by ETH, ALD, or e-Cig stimulation increased the expression of IRE1α and pASK1proteins, leading hPAEpiC to undergo severe stress. By lowering BI-1 protein expression, ETH, ALD, or e-Cig (1.8% nicotine) stimulation promoted toxic ER-Ca^2+^ levels (Fig. 3). P2X7r inhibition by A80 restored the ER-Ca^2+^ levels and reduced the expression of IRE1α and pASK1proteins, ensuring lung epithelial cell survival.

EVs released from injured cells differ significantly in their structure and function. EVs carry and transport unique biomolecules depending on the disease conditions, making them perfect biomarkers [71]. In lung carcinoma cells, nicotine stimulation increases EV number and transforms EVs’ morphology with an altered miRNA profile[72]. Active inhalation of nicotine-containing e-Cig vape increased circulating EV number, shed by endothelial cells and loaded with proinflammatory CD40 markers [73]. In patients, nicotine consumption aggravates the spread of atherosclerotic lesions, potentially via EVs containing miR-21–3p cargo[74]. Likewise, liver injury inflicted by alcohol abuse also exaggerates EV release, carrying inflammatory signaling molecules (NFκB, TLR4, IL-1 receptors, caspase-1) into the circulation [75]. In the brain, cocaine-induced oxidative stress weakens mitochondrial membrane potential, forcing the mitochondria to rupture and release their contents via EVs[76, 77].

P2X7r activation by eATP and/or NAD + molecules promotes P38-MAPK-facilitated cytoskeletal restructuring in macrophages, resulting in EV release[78]. However, under normal conditions, intercellular ATP and NAD^+^ levels remain low for P2X7r activation. Chronic alcohol exposure in humans stimulates inflammasome activation in the liver and brain, followed by tissue damage, resulting in substantial eATP release[79]. Once released, eATP acts as an endogenous mediator and enhances EV release[80, 81]. eATP that are endocytosed into EVs also mediate actin rearrangement and influence EV size, shape, and adhesion properties[82]. According to our NTA data, hPAEpiC stimulated with ETH-, ALD-, or e-Cig (1.8% nicotine)-conditioned media, generated more EVs (2-fold to 3-fold increase) with larger size than unexposed hPAEpiC (Fig. 4). Pretreatment with A80 reverted the EV numbers and size to those shed by untreated hPAEpiC.

In the mouse brain, cocaine-induced inflammation promotes the release of small EVs (exosomes) loaded with mtDAMPs, including misfolded mito-proteins, eATP, ROS, and degraded mtDNA[83]. When released, these mtDAMPs can activate numerous proinflammatory autocrine and paracrine signaling in recipient cells, producing several inflammation-associated diseases[84]. In our studies ETH, ALD, or e-Cig (1.8% nicotine) exposure increased eATP cargo in EVs. dPCR analyses showed the large quantities of mtDNA embedded in the lung epithelial EVs (Fig. 5), which can act as mtDAMPs. P2X7r inhibition in lung cells exposed with ETH-, ALD-, or e-Cig-conditioned media reversed EV cargo, confirming the role of the P2X7r pathway on lung-EV release and on its cargo.

In addition to their unique cargo-carrying capacity, EVs also carry surface ligands/receptors, allowing EVs to target other cells[85]. Once attached on the recipient cell, EVs transmit signals via receptor-ligand interaction or internalized by endocytosis or fused with the recipient cell membrane, delivering their cargo into its cytosol, thereby altering the functional state of the recipient cell[86]. In human macrophages, P2X7r stimulation by eATP promotes inflammation and release of EVs loaded with IL-1b and P2X7r[87, 88]. Similarly, chronic inflammatory responses seen in diabetic and COVID19 patients resulted in P2X7r release into the circulation[89, 90], most likely through EVs. Our studies demonstrated significant quantities of circulating P2X7r in the lung epithelial cell media and EVs with greater P2X7r expression on their surface (Fig. 6) against ETH, ALD, or e-Cig (1.8% nicotine) stimulation. P2X7r can further stimulate inflammation in recipient cells directed by NLRP3 activation[91].

Interestingly, we detected a variety of cargoes (eATP, mtDNA) in hPAEpiC-EVs that can act as mtDAMPs, which can trigger NLRP3 inflammasome mediated BBB damage[92, 93]. P2X7r found on EVs is known to activate NLRP3-mediated Ca^2+^ accumulation in bone marrow cells [94]. In this cotext, we assessed the paracrine signaling efficacy of biomolecules detected in lung EVs by incubating BMVECs (cells constituting BBB) with lung epithelial cell-spent media and freshly isolated lung EVs. In both experiments, in line with circulating eATP (Fig. 5) and P2X7r levels (Fig. 6), we noticed a significant amount of Ca^2+^ accumulation in hBMVECs (Fig. 7), whereas spent media and EVs derived from epithelial cells pretreated with A80 displayed lower Ca^2+^ levels, endorsing the paracrine signaling induced by alcohol or nicotine-containing e-Cig in hPAEpiC and BMVECs. Notably, recombinant P2X7r added to hBMVECs resulted in the similar functional changes as ETH, ALD, or e-Cig (1.8% nicotine) executed (Fig. 7B).

In all our experiments, we exposed hPAEpiC with e-Cig (0% nicotine)-conditioned media, as our earlier studies with nicotine-free e-Cig vape produced pathological effects on mouse brain and lung tissues [3]. Airway epithelail cells exposed with nicotine-free e-Cig vape also increased the IL-6 levels[95] and limited the oxygen levels in circulation[96]. Some studies in patients with asthma have also shown that nicotine-free e-liquids, made of high grade, contaminant-free mixture of propylene glycol and glycerol, did not impact lung function [97]. In this report, nicotine-free e-Cig media increased P2X7r levels marginally, resulting in a partial increase in intracellular Ca^2+^ levels, which did not affect any of our functional assays.

## Conclusion

The current study demonstrated the harmful effects of e-Cig (1.8% nicotine), ETH, and its main metabolite ALD on mitochondrial function in hPAEpiC, which form an alveolar barrier. Ca^2+^ accumulation promoted by drugs of abuse stimulates ER stress, increase EV release bolstering greater eATP, P2X7r cargo transport. Mito-stress induced by ETH and e-Cig (1.8% nicotine) stimulation induce the mitochondrial membrane damage, letting mtDNA escape through EVs. A variety of cargo detected in hPAEpiC-EVs act as potential stimulants of inflammation and trigger functional changes in BMVECs, indicative of BBB injury. These observations also demonstrate the mechanisms of distant organ injury by e-Cig or alcohol. Similar functional changes exerted by recombinant P2X7r in BMVECs, confirms its role as a paracrine signaling molecule for the first time. Inhibition of P2X7r diminished all pathological effects caused by ETH, ADH or e-Cig (1.8% nicotine) in hPAEpiC. We will continue to test the impacts of abusive drugs on P2X7r expression and its extracellular sheding in the mouse model and test P2X7r paracrine siganling in the BBB damage.

## Figures and Tables

**Table 1:** ETH, ALD, and e-Cig (1.8% nicotine) exposure increased the size and numbers of shed lung epithelial EVs, and P2X7r inhibition brought them to control levels. (A) Overnight stimulation of hPAEpiC with ETH, ALD, and e-Cig (1.8% nicotine)-conditioned media increased the EV particle size by 20–30%. The EV number was increased by 2-fold after ETH exposure and 3-fold after ALD or e-Cig (1.8% nicotine) exposure. In contrast, A80 pretreatment decreased the size and number of EVs. Data is presented as mode ± standard error) (n = 3).

S.No	Treatment	Particle size ± standard error	Concentration (particles/mL)
1	Nontreated (NT)	136.8 ± 4.7 nm	3.04 × 10^8^
2	ETH	149.5 ± 4.4 nm	6.11 × 10^8^
3	ETH + A80	127.2 ± 5.7 nm	4.88 × 10^8^
4	ALD	155.3 ± 3.5 nm	8.71 × 10^8^
5	ALD + A80	126.7 ± 3.6 nm	6.11 × 10^8^
6	1.8% e-Cig	153.8 ± 6.8 nm	8.10 × 10^8^
7	1.8% e-Cig + A80	138.7 ± 2.5 nm	6.08 × 10^8^
8	0% e-Cig	145.8 ± 5.6 nm	4.16 × 10^8^
9	0% e-Cig + A80	138.9 ± 5.4 nm	3.85 × 10^8^
10	A80	134.0 ± 4.3 nm	4.01 × 10^8^

## Abbreviations

ETH: Ethanol
ALD: Aldehyde
e-Cig: electronic cigarette
hPAEpiC: human pulmonary alveolar epithelial cells
hBMVECs: human brain microvascular endothelial cells
mtDNA: mitochondrial DNA
eATP: extracellular ATP
NLRP3: NLR family pyrin domain containing 3
P2X7r: P2X purinergic receptor 7
TRPV1: Transient receptor potential vanilloid 1
FCCP: carbonyl cyanide p-trifluoro methoxyphenylhydrazone
OXPHOS: oxidative phosphorylation
SRC: spare respiratory capacity
ROS: Reactive oxygen species
RNS: Reactive nitrogen species
ER: endoplasmic reticulum
UPR: unfolded protein response
mtDAMPs: mitochondrial damage-associated molecular patterns
